# Importance of Wetlands Management for West Nile Virus Circulation Risk, Camargue, Southern France

**DOI:** 10.3390/ijerph110807740

**Published:** 2014-08-04

**Authors:** Sophie Pradier, Alain Sandoz, Mathilde C. Paul, Gaëtan Lefebvre, Annelise Tran, Josiane Maingault, Sylvie Lecollinet, Agnès Leblond

**Affiliations:** 1 INRA, UR346 Epidémiologie Animale, Saint Genès Champanelle F-63122, France; E-Mail: agnes.leblond@vetagro-sup.fr; 2 INRA, UMR1225, IHAP, Université de Toulouse, INP-ENVT, Toulouse F-31076, France; E-Mail: m.paul@envt.fr; 3 Centre de Recherche pour la Conservation des Zones Humides Méditerranéennes, Fondation Tour du Valat, Arles F-13200, France; E-Mails: sandoz@tourduvalat.org (A.S.); lefebvre@tourduvalat.org (G.L.); 4 UFR Sciences, Aix-Marseille Université, Marseille F-13003, France; E-Mail: alain.sandoz@univ-amu.fr; 5 CIRAD, UPR Animal et Gestion Intégrée des Risques (AGIRs), Montpellier F-34398, France; E-Mail: annelise.tran@cirad.fr; 6 CIRAD, UMR Territoires Environnement Télédétection et Information Spatiale (TETIS), Montpellier F-34398, France; 7 ANSES, Maisons-Alfort Laboratory for Animal Health, UMR1161 Virologie, INRA, ANSES, ENVA, Maisons-Alfort F-94703, France; E-Mails: slecollinet@vet-alfort.fr (S.L.); josiane.maingault@anses.fr (J.M.); 8 Equine Department, Vetagrosup, Marcy L’Etoile F-69280, France

**Keywords:** West Nile, France, wetlands, spatial analysis, risk factor, vector-borne diseases, remote sensing

## Abstract

To assess environmental and horse-level risk factors associated with West Nile Virus (WNV) circulation in Camargue, Southern France, a serosurvey was conducted on non-vaccinated horses (*n* = 1159 from 134 stables) in 2007 and 2008. Fifteen Landsat images were examined to quantify areas with open water and flooded vegetation around sampled horses. Mean percentages of areas of open water and flooded vegetation, as well as variations in these percentages between 3 periods (November to February = NOT, March to July = END and August to October = EPI), were calculated for buffers of 2 km radius around the stables. Results of the final logistic regression showed that the risk of WNV seropositivity in horses decreased with their date of acquisition and age. Results also demonstrated the significant role of environmental variables. Horse serological status was associated with variations of open water areas between the NOT (November to February) and END (March to July) periods, as well as between END and EPI (August to October). WNV spillover was found more intense in areas where water level decreased strongly from winter to spring and from spring to summer.

## 1. Introduction

West Nile Virus (WNV), a flavivirus belonging to the Japanese encephalitis virus serocomplex, is transmitted in natural cycles between mosquitoes, (mainly the genus *Culex*), and wild birds. Horses and humans are incidental and dead-end hosts, but can develop severe neurological disorders [1]. About 20% of infected horses develop a disease and, in the “Old World”, horses are considered as sentinels of WNV circulation in some areas as the mortality in birds is very low [2].

Recently, the number of WNV cases reported in horses and humans in Europe has increased dramatically. Large outbreaks of increased clinical severity have been reported in parts of Russia, Southern and Eastern Europe [3,4,5]. Until 2010, most of these outbreaks were caused by strains of WNV lineage 1. From 2010 onwards, lineage 2 emerged as the major virus responsible for European WNV outbreaks [6]. Despite the frequent detection of WNV in neighboring countries, no equine case of WNV has been detected in France since 2006 [7]. However, serological studies conducted from 2005–2007 in wild birds in Camargue showed that two juveniles sampled in 2006 were seropositive and that another seroconverted in 2007, thus revealing circulation of the virus in Camargue during this period [8]. In another investigation, conducted in this area during 2009, a high titer of WNV-neutralizing antibodies was found in a second year magpie (*Pica pica*), indicating its recent exposure to the virus [9]. Thus, despite evidence of the regular circulation of WNV in wild birds in Camargue, no clinical case has been reported in this area since 2004. Furthermore, in north east Italy in 2008, cases of WNV lineage-1 were observed in horses in a stable close to the Po river, about 50 km from the Comacchio marsh area in Ferrara province [10]. Since 2008, outbreaks have been observed almost every year and the area is now considered endemic [6]. So, although the ecological situations in Camargue and Emilia-Romagna are seemingly comparable, the disease situations are different. We hypothesize that the management of Camargue wetlands could play a key role in the absence of outbreaks in this area during recent years.

WNV transmission requires competent vectors, receptive hosts, and environmental parameters which allow contact between the vectors and the different hosts. In southern France, WNV is transmitted by* Culex modestus* Ficalbi and *Culex pipiens* Linnaeus [11]. The Rhône Delta is known for its landscape of wetlands and its wealth of different bird species. Livestock species include horses, cows and sheep. The Camargue hosts nearly 100,000 permanent inhabitants and, in summer, the number of people increases due to tourism [12]. It is also a region where mosquito populations are very abundant. Recent observations have shown that environmental changes, mainly resulting from anthropogenic practices, have had an impact on inter-annual variations in the wetlands and consequently on the abundance of mosquitoes [12]. Water is provided either by rainfall or by a very tight canal network diverted from the river Rhone. Water management is under the responsibility of individual field owners and dependent on its various uses (grazing, rice culture, hunting reserves,* etc.*) [13]. We assumed that this specific management of water resources could play a role in the circulation of WNV in the area. In this context, a sero-ecological study was conducted in Camargue to assess the relative importance of environmental variables related to wetlands and their variations, on the WNV circulation pattern observed in horses. 

## 2. Experimental Section 

### 2.1. Study Area

**Figure 1:**
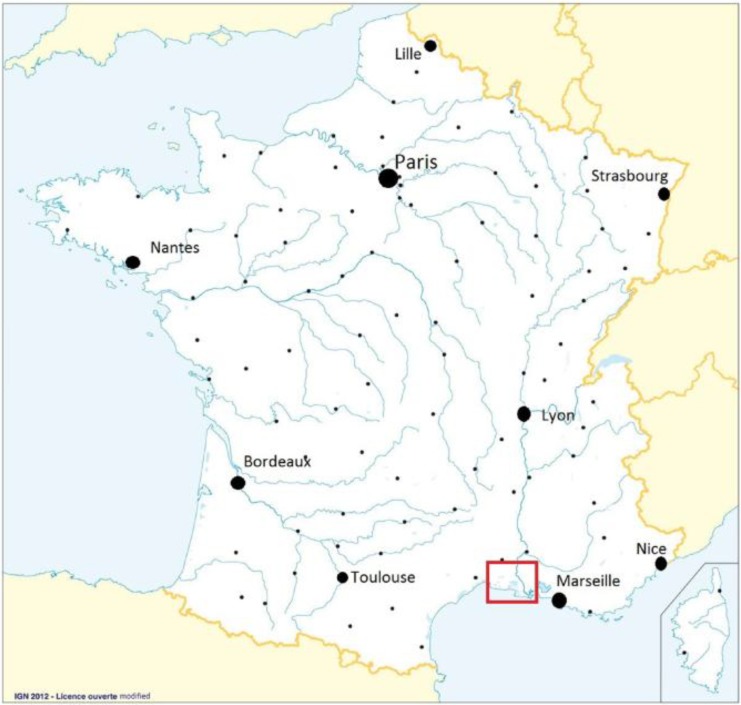
Localization of the study area in France (in the red rectangle).

The Camargue is located in the Rhone river delta near the Mediterranean coast in south-eastern France (Figure 1). From an ecological point of view, this region is an important wetland presenting diversified environments in which the dry areas are continuously irrigated by canals, ditches and wetlands [14]. More than 300 bird species have been observed in this area [15] and large mosquito populations are present due to the great abundance and diversity of mosquito-breeding sites [16]. Rice is the main crop cultivated in Camargue. The rice fields are filled with water from April to August, after which the water is drained and the rice harvested [16]. In 2000, the equine population in Camargue was estimated by the French Agricultural Census of livestock at around 7,000 individuals [17]. 

### 2.2. Field Sampling and Laboratory Analysis

The target population included all horses living in Camargue, but actually corresponded to the horses recorded by veterinarians in the study area and non-vaccinated against WNV. In 2007–2008, no WNV vaccine was authorized yet for horses in France. Stables were randomly selected from a database of 201 stables which had been created and maintained since the 2000 WNV outbreak in Camargue [18]. All those stables were targeted in the present study, the objective being to sample on average 10 horses per stable. All the stables were georeferenced; they were defined as the place where horses spend most of the year, especially during the season of mosquito activity. Depending on the type of stable, it could correspond to a riding centre or a pasture for example. Two horse sampling campaigns were conducted (May to July 2007 and April to June 2008) before the known period of WNV transmission to equids (*i.e.*, late summer) [5]. At the same time, individual characteristics of the sampled horses were also recorded by means of a questionnaire. 

After blood collection, the serum was separated by gentle centrifugation and stored at −20 °C. Sera were processed and tested for anti-WNV antibodies using an enzyme-linked immunosorbent assay (ELISA) (ID Screen^®^ West Nile Competition, Montpellier, France) in the European Union Reference Laboratory (EU-RL) for Equine Diseases (ANSES, Maisons-Alfort, France). ELISA-positive results were assumed to be WNV-specific due to the absence of other flaviviruses in the region in 2007–2008. 

### 2.3. Environmental Data

Fifteen Landsat Images, corresponding to the study area and periods of interest, were freely acquired from the Landsat 7 Archive Earth Explorer website [19]. Landsat sensors cover an area of 185 km × 185 km with a 30 m spatial resolution. The mid-infrared channel can be used to discriminate between open water and vegetation. Since July 2003, the Landsat 7 images have been damaged. Uninformed bands cross images from east to west. To circumvent this problem, we used a generalization process with GeoImage^TM^ software.

Pixels of the images were classified into three land cover classes: open water (coastal lagoons, marshes,* etc.*), flooded vegetation (rice fields, reed beds,* etc.*) and other, by means of a supervised classification with the GeoImage^TM^ software.

Based on expert knowledge, the resulting classifications were grouped into three periods. The mean percentages of open water and flooded vegetation areas on each image for all stables, and finally the averages of these mean percentages were measured for each period (Table 1). Depending on the different levels of mosquito activity in the Camargue region, these periods of interest were named: “NOT” (for “NOT at risk period”, November to February), “END” (for “period at risk of ENDemic WNV circulation”, March to July) and “EPI” (for “period at risk of EPIdemic WNV circulation”, August to October). Areas of open water and flooded vegetation were calculated using a Geographic Information System (ESRI^®^, ArcGIS™ 9.3) software in 2-, 3- and 5-km radius buffers around the stables for the three periods. This buffer radius was chosen on the basis of the known flight range of *Culex spp.* [20] and regular movements of horses around the centroid of the buffer, e.g., for grazing. 

**Table 1:** Groups of Landsat Images depending on the level of mosquito activity in the Camargue region, Southern France.

Groups	Dates of Images	
Very low level of mosquito activity = “NOT”	2006:	10 December
		26 December
	2007:	11 January
		27 January
		13 December
	2008:	15 February
Medium level of mosquito activity = “END”	2007:	16 March
		17 April
	2008:	2 March
		22 June
		8 July
High level of mosquito activity = “EPI”	2006:	20 August
	2007:	8 September
		24 September
	2008:	9 August

### 2.4. Statistical Analysis

The variables associated with equine seropositivity were identified by applying generalized linear mixed models. The binomial response variable was the individual serological status. Clustering of horses in stables was taken into account by introducing the stable as a random effect in this logistic model. The risk factors considered included five individual variables (breed, age, gender, activity and date of acquisition in the stable) as well as twelve environmental variables:

(1) The log-transformed mean percentages of the area corresponding to open water or flooded vegetation in buffers around the stables, during the three time periods NOT, END and EPI (6 variables).
(2) The differences between these percentages between the three periods: NOT-END, END-EPI and NOT-EPI (6 variables). For example, for a given location, END-EPI for open water represents the mean percentage of the area of open water during the END period minus the mean percentage of the open water area during the EPI period and expresses the variation in open water areas between the endemic and epidemic periods, within a buffer of 2 km radius around the stables. These differences were categorized in tertiles.

Correlation between each explanatory variable was first examined using the Pearson correlation test. If two variables showed strong correlation (correlation coefficient > 0.8), only the variable showing the lowest p-value in the univariate regression model was entered into the multivariate process. All of the putative risk factors were screened in univariate regression models. Variables that were significant (*p* ≤ 0.20) in the univariate phase were then retained to enter into the full multivariate starting model. Stepwise selection of the final model was carried out based on the Akaike criteria (AIC) [21].

Epidemiological data often display spatial autocorrelation,* i.e.*, locations close to each other exhibit values that are more similar than those further apart [22]. Continued presence of this pattern in the residuals of a statistical model based on such data can result in biased parameter estimates [23]. However, if the spatial dependence in the response variable is completely explained by the pattern in the exposure variable, no spatial autocorrelation should be present in the regression residuals and regression will produce correct effect estimates and confidence intervals [22]. Any not-accounted-for spatial correlation in the residuals of the logistic model was identified by applying the described Monte Carlo method [24] used in previous studies [25]. Goodness-of-fit of the final model was assessed by computing the area under the curve (AUC) of the receiver operating characteristic plots. All analyses were performed using R software version 3.0.2. 

## 3. Results and Discussion

### 3.1. Results

#### 3.1.1. Serological Results

**Figure 2:**
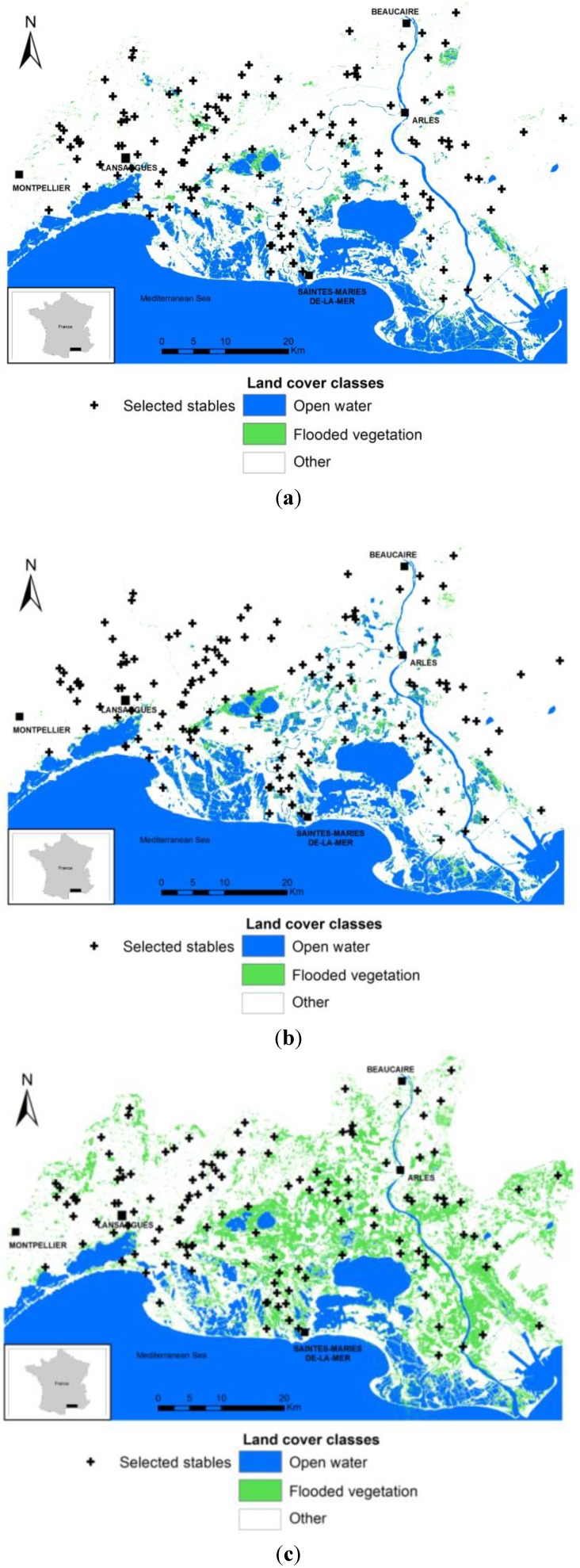
(**a**) Classification of a Landsat Image of the NOT period (11 January 2007); (**b**) Classification of a Landsat Image of the END period (17 April 2007); (**c**) Classification of a Landsat Image of the EPI period (8 September 2007).

1159 serum samples were collected from horses in a total of 134 stables. The percentage of sampled horses in stables varied from 7% to 100% (mean = 50.7%), depending on the owner’s availability and the accessibility and docility of the horses. In total, 142 horses out of 1159 (overall observed prevalence rate = 12.3%, 95% CI: 10.1–14.4), belonging to 61 stables, were found seropositive for anti-WNV antibodies. Figure 2 shows the spatial distribution of stables with WNV seropositive or seronegative horses. 

#### 3.1.2. Univariate Analysis

**Figure 3:**
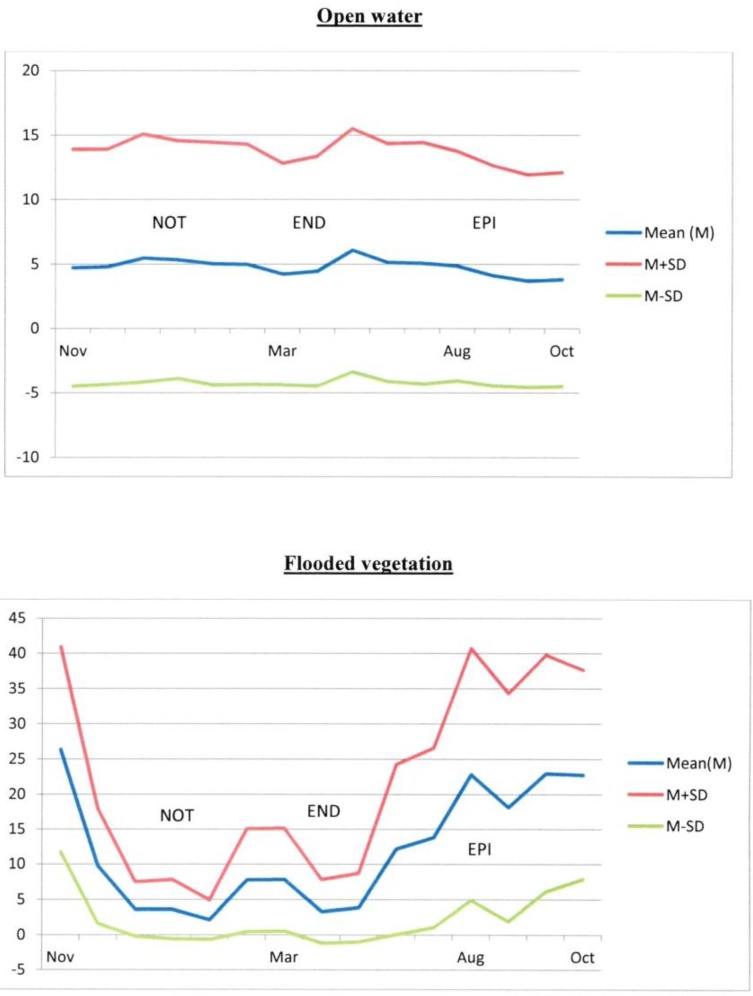
Variations in mean percentages of open water and flooded vegetation areas in buffers of 2-km radius around stables in the study zone, during the study period (SD = Standard Deviation).

Figure 3 and Table 2 show the variations in the mean percentages of areas corresponding to open water and flooded vegetation in the study zone during the study period in buffers of 2 km-radius. Larger areas of open water are found during the NOT period, then the areas decrease slightly during the END period. A notable increase is apparent in April when the rice fields are irrigated. Finally, the open water areas fall to their lowest level during the EPI period. For the flooded vegetation, the highest levels are observed during the EPI period. A slight decrease in the areas of flooded vegetation was measured between the NOT and END periods.

All 17 candidate risk factors were associated with WNV seropositivity at *p* ≤ 0.20 (Table 2 and Table 3) and were selected for inclusion in the multivariate modelling process. As results obtained with buffers of 2 to 5 km gave similar results (data not shown), we retained only variables corresponding to buffers of 2-km radius for further analysis.

**Table 2:** Distribution and results of the univariate analysis for the environmental explanatory variables calculated in buffers of 2 km-radius around each stable and tested for their association with the serological status of WNV in equids in Southern France, 2007–2008.

Variable	Mean (Min; Max)	Adjusted Odds-Ratio	95% Confidence Interval	*p*-value
Mean percentage (%) of open water area ^a^:				
NOT	5.025 (0.0; 52.70)	2.07	[1.47–2.92]	<0.001
END	4.962 (0.0; 50.83)	2.16	[1.53–3.05]	<0.001
EPI	4.083 (0.0; 50.67)	1.91	[1.35–2.7]	<0.001
Mean % of flooded vegetation area ^a^:				
NOT	8.831 (0.88; 31.84)	2.96	[1.46–6.02]	0.003
END	8.170 (0.09; 35.98)	3.35	[1.94–5.79]	<0.001
EPI	21.612 (1.06; 64.61)	2.44	[1.42–4.2]	0.001
Differences in mean % of open water area (e.g.,: NOT-END = mean% NOT − mean% END) :				
NOT-END	0.064 (−3.70; 3.88)			
[−3.70; 0.01]		NS	[0.29–0.83]	0.008
[0.01; 0.14]		0.49		
[0.14; 3.88]		NS		
END-EPI	0.878 (−1.03; 8.01)			
[−1.03; 0.0]		NS		
[0.0; 0.88]		NS		
[0.88; 8.01]		3.83	[1.71–8.56]	0.001
NOT-EPI	0.943 (−1.73; 10.50)			
[−1.73; 0.04]		NS		
[0.04; 0.78]		NS		
[0.78; 10.50]		2.15	[1.35–3.43]	0.001
Differences in mean % of flooded vegetation area:				
NOT-END	0.661 (−13.15; 11.92)			
[−13.15; −0.08]		NS	[0.38–0.9]	0.015
[−0.08; 2.88]		0.59		
[2.88; 11.92]		NS		
END-EPI	−13.440 (−46.43; −1.06)			
[−46.43; −17.55]		NS		
[−17.55; −5.11]		0.49	[0.31–0.79]	0.003
[−5.11; −1.06]		0.58	[0.38–0.88]	0.011
NOT-EPI	−5.747 (−48.01; 3.97)			
[−48.01; −16.97]		NS		
[−16.97; −2.34]		NS		
[−2.34; 3.97]		0.45	[0.27–0.76]	0.003

^a^ before log-transformation.

**Table 3:** Distribution and results of the univariate analysis for the horse-level explanatory variables tested for their association with the serological status of West Nile Virus in equids in Southern France, 2007–2008.

Variable	Counts	Categories	% Seropositive	Adjusted Odds-Ratio	95% Confidence Interval	*p*-value
Horse gender	1157 ^a^					
	133	Stallion	0.4	NS		
	673	Gelding	9.0	4.71	[1.88–11.82]	<0.001
	351	Mare	2.9	2.67	[1.02–6.99]	0.046
Horse breed	1159^ a^					
	405	Other	2.8	NS		
	632	Camargue	9.1	2.21		
	122	Iberic	0.4	NS	[1.45–3.37]	<0.001
Horse activity	1156^ a^					
	297	Other	2.9	NS		
	179	Breeding	2.0	NS		
	276	Livestock cutting	4.3	2.11	[1.29–3.48]	0.003
	404	Riding school	3.1	NS		
Horse birth date	1136^ a^					
	185	≤1990	3.6	24.85	[3.35–184.39] [1.72–92.48] [1.52–81.74]	<0.001
	413	[1991–1997]	4.4	12.61		<0.001
	446	[1998–2004]	4.2	11.13		<0.001
	92	≥2005	0.1	NS		
Date of horse acquisition	1122^ a^					
	384	≤1999	7.0	NS		
	422	[2000–2004]	4.0	0.45	[0.3–0.67]	<0.001
	316	≥2005	1.2	0.16	[0.09–0.3]	<0.001

^a^ Missing values excluded; NS = non significant.

#### 3.1.3. Multivariate Analysis

The variable “gender” was excluded from the multivariate analysis because it was highly correlated with “activity” and was of less biological interest in the analysis than other individual variables. 

The mean percentages of open water areas during the three periods, as mean percentages of flooded vegetation areas during the periods END and EPI and variations in the flooded vegetation areas between the periods END-EPI and NOT-EPI, were highly correlated. 

So the full starting model included 12 variables:

(1) Age, breed, date of acquisition in the stable, activity,
(2) Mean percentage of open water area during the EPI period and mean percentages of flooded vegetation during the NOT and END periods,
(3) All the differences in open water and flooded vegetation areas except the difference in flooded vegetation between the NOT and EPI periods.

The final model (Table 4) included four explanatory variables. WNV horse seropositivity was influenced by the date of horse acquisition in the stable and its age. In addition, the odds of seropositivity increased for areas characterized by a strong reduction of open water around stables between the NOT and END periods, and between END and EPI periods.

**Table 4:** Results of the final logistic regression model for the serological status of West Nile virus in equids in Southern France, 2007–2008.

Variable	Adjusted Odds-ratio	95% Confidence interval	*p*-value
Horse birth date:			
≤1990	1		
[1991–1997]	0.65	0.36–1.17	0.147
[1998–2004]	0.97	0.49–1.91	0.921
≤2005	0.09	0.01–0.93	0.043
Date of horse acquisition:			
≤1999	1		
[2000–2004]	0.50	0.28–0.87	0.014
≥2005	0.27	0.12–0.62	0.002
Differences in mean % of open water area	(2 km buffers)		
NOT-END			
[−3.70; 0.01]	1		
[0.01; 0.14]	1.78	0.62–5.13	0.285
[0.14; 3.88]	2.33	1.03–5.28	0.042
END-EPI			
[−1.03; 0.0]	1		
[0.0; 0.88]	1.10	0.33–3.61	0.878
[0.88; 8.01]	5.53	1.53–20.01	0.009

Computation of the observed residuals of this model by Monte Carlo method did not reveal any evidence of an unaccounted spatial pattern in the residuals. The AUC of the receiver operating characteristic plots calculated for the final model was 0.88, indicating a reasonable discriminatory ability [26].

### 3.2. Discussion

Our findings, based on remote sensing, questionnaire and WNV equine serological data, provide new information about the relationship between variations of wetlands and WNV circulation in Southern France, as well as about the intrinsic determinants associated with horse seropositivity. These results could be useful to the health authorities to improve wetlands management and reduce the risk of disease transmission in mammalian hosts. Monitoring key environmental parameters could be helpful to forecast periods at risk for disease transmission and to decide when preventive measures should be implemented. 

This study was original because it was based on a very large number of free satellite images which were used to determine fine variations in areas of open water and flooded vegetation over a year throughout the study zone. To our knowledge, this type of study has never been performed on WNV. In addition, the large number of images and their spatial coverage made it possible to highlight wet areas which are only temporarily flooded such as rice fields, and to produce a uniform classification throughout the study area.

The study had some limitations that need to be discussed before conclusions are drawn. The present study was based on a large sample of horses which, at first glance, can be considered representative of the equine population in the study area. Indeed the Camargue breed and livestock cutting horses, which are very specific to the study area, were well represented in our sample. However, as the database used for stable sampling corresponded to those horses registered in veterinary clinics, we cannot exclude a selection bias due to the exclusion of horses which were not registered. This may have influenced the results for WNV prevalence in horses, but not the spatial analysis of risk factors, provided that the selection bias was homogeneously distributed across the study area. Getting a better insight into the size and geographic distribution of the equine population should make it easier to design improved sampling schemes for future studies. Another potential limit in the study concerning the serological analysis was the use of the ELISA test which is known to cross react with antibodies against other flaviviruses. To verify our results, WNV neutralization tests have been performed on about 25% of the sera found positive in competition ELISA (36/142; tests performed at ANSES, Maisons-Alfort). Every sera (36) were found positive for WNV neutralizing antibodies, indicating that the corresponding horses had been infected by WNV. So WNV appears to be the most probable flavivirus in the Camargue region, at the time of the survey (2007–2008). 

The present study furthers our current understanding of the environmental conditions which may favor WNV circulation in Southern France. It highlighted the positive association between a greater decrease in open water areas (such as coastal lagoons and marshes) around the stables, between the NOT (November to February) and END (March to July) periods and between END and EPI (August to October) with an increase in WNV spillover in horses in Camargue. These results suggest that WNV spillover was more intense in areas with a high number of humid areas in winter and spring and a high consecutive decrease in the water level during spring and summer, respectively. It is likely that these variations influence the presence and size of the WNV vector populations in the horses’ surroundings. Further studies need to be implemented and the results compared with investigations in other WNV-endemic areas such as Italy, before conclusions can be drawn. 

To explain the association between horse seropositivity and a greater decrease in the level of open water between the END and EPI periods, we suggest that a large area of open water is needed in March to July, during the endemic cycle between birds and mosquitoes, and then a smaller open water area close to the horses during the epizootic cycle, where wild birds will gather in large numbers at the same time that mosquitoes are very active and aggressive. All the required criteria are then assembled to permit full-speed functioning of the WNV epidemiological cycle, which consequently facilitates the transmission to horses by bridge vectors. 

The hypothesis had already been put forward in early 2000 that a mild winter, followed by a dry spring and summer, associated with heavy rains in August, could favor the emergence of WN disease in the USA [27]. These conditions allow the survival and proliferation of large populations of mosquitoes and favor the congregation of hosts, mammals and birds during the period of transmission [28]. The spatial-temporal variability of human WN cases and transmission of WNV to sentinel chickens were also associated with the spatial-temporal variability of drought and land surface wetting in southern Florida [28].

The role of open water in WNV circulation is still under debate and previous investigations have provided contradictory results. However, the influence of variations had not yet been studied. When the proposed model was used in 2002 in a study in North Dakota, USA, no association between the presence of permanent water bodies and WNV occurrence in horses was found [29]. Another study conducted in Canada in 2003 showed that high-risk areas for WNV infection in horses corresponded to areas where the percentage covered by open water was low [30]. In contrast, a study carried out in Texas in 2002–2004, showed that the occurrence of WNV clinical cases in horses was more frequent in the vicinity of the Brazos River watershed and lakes [31]. Our study showed that wetland variations are more important in WNV spillover in horses than the wetland areas alone, which can perhaps explain these contradictory results. 

## 4. Conclusions

This study showed that besides horse-level risk factors, the risk of WNV horse seropositivity was associated with increasing variations in the open water areas around stables between particular periods of the year. These results could help to target risk-based surveillance on specific areas and at specific periods of the year. 
